# The multiple PDZ domain protein Mpdz/MUPP1 regulates opioid tolerance and opioid-induced hyperalgesia

**DOI:** 10.1186/s12864-016-2634-1

**Published:** 2016-04-29

**Authors:** Robin Donaldson, Yuan Sun, De-Yong Liang, Ming Zheng, Peyman Sahbaie, David L. Dill, Gary Peltz, Kari J. Buck, J. David Clark

**Affiliations:** Department of Computer Science, Stanford University, Stanford, CA USA; Anesthesiology Service, Veterans Affairs Palo Alto Health Care System, 3801 Miranda Ave., Anesthesiology, 112A, Palo Alto, CA 94304 USA; Department of Anesthesiology, Perioperative and Pain Medicine, Stanford University School of Medicine, Stanford, CA USA; Department of Behavioral Neuroscience, Oregon Health and Science University, Portland, OR USA

**Keywords:** Opioid analgesics, Drug tolerance, Gene mapping, Synaptic plasticity

## Abstract

**Background:**

Opioids are a mainstay for the treatment of chronic pain. Unfortunately, therapy-limiting maladaptations such as loss of treatment effect (tolerance), and paradoxical opioid-induced hyperalgesia (OIH) can occur. The objective of this study was to identify genes responsible for opioid tolerance and OIH.

**Results:**

These studies used a well-established model of ascending morphine administration to induce tolerance, OIH and other opioid maladaptations in 23 strains of inbred mice. Genome-wide computational genetic mapping was then applied to the data in combination with a false discovery rate filter. Transgenic mice, gene expression experiments and immunoprecipitation assays were used to confirm the functional roles of the most strongly linked gene. The behavioral data processed using computational genetic mapping and false discovery rate filtering provided several strongly linked biologically plausible gene associations. The strongest of these was the highly polymorphic *Mpdz* gene coding for the post-synaptic scaffolding protein Mpdz/MUPP1. Heterozygous *Mpdz +/−* mice displayed reduced opioid tolerance and OIH. *Mpdz* gene expression and Mpdz/MUPP1 protein levels were lower in the spinal cords of low-adapting 129S1/Svlm mice than in high-adapting C57BL/6 mice. Morphine did not alter *Mpdz* expression levels. In addition, association of Mpdz/MUPP1 with its known binding partner CaMKII did not differ between these high- and low-adapting strains.

**Conclusions:**

The degrees of maladaptive changes in response to repeated administration of morphine vary greatly across inbred strains of mice. Variants of the multiple PDZ domain gene *Mpdz* may contribute to the observed inter-strain variability in tolerance and OIH by virtue of changes in the level of their expression.

## Background

The US Centers for Disease Control (CDC) has declared opioid prescribing to be at epidemic levels [1]; use of these drugs primarily for the treatment of chronic pain has increased exponentially over the past 15 years in the US and many other Western nations [2]. Unfortunately, opioid therapy is often accompanied by opioid dose escalation, loss of treatment effect, progression of pain complaints and disability. For example, more than 1/3^rd^ of chronic pain patients double their oral opioid doses in the first few years of treatment [3], and those treated with intrathecal opioids experience 12–17 % increases in opioid doses each year on average [4]. Moreover, clinical studies have shown greater pain sensitivity in chronic pain patients treated with opioids with changes proportional to the duration of therapy and the doses used [5]. Equally concerning, opioid dose escalation either in the setting of acute injury or when used to provide analgesia for chronic pain patients is associated with increased risks of side effects, abuse, overdose and death [6]. These problems involving loss of treatment effect and consequent dose escalation are believed to be caused in large part by maladaptations such as tolerance and the related phenomenon of opioid-induced hyperalgesia (OIH) [7]. We have no clinically available strategies effective in preventing or reversing these changes.

Genetic approaches to understanding opioid maladaptations offer the advantage of taking a hypothesis-free approach to the identification of underlying mechanisms. Laboratory studies using inbred mice demonstrate that analgesic tolerance, physical dependence and opioid-induced hyperalgesia all display a very large degree of heritability (>60 %) [8–11]. Likewise, numerous studies conducted in mice have identified quantitative trait loci (QTLs) and individual genes linked to specific maladaptations such as tolerance [12], dependence [13, 14] and OIH [9, 15]. In the case of the β2-adrenergic receptor linked to OIH and the 5-HT3 receptor linked to physical dependence, the murine genetic findings were confirmed in small translational trials in humans [13, 16].

Haplotype-based computational genetic mapping (HBCGM) has recently advanced to the point that whole-genome sequence data can be used to identify genetic factors affecting drug responses [17]. Using this approach, we recently found that the *Dcc* gene affects OIH and tolerance [18]. However, highly polymorphic genes have a relatively high probability for matching unrelated phenotypes by HBCGM due to the large number of haplotype blocks that can be formed within the gene. This effect can be particularly problematic when multiple different phenotypes are evaluated. Hence, a more accurate ranking of genes can be obtained if we correct for the false discovery rate (FDR) of each gene [19]. In this set of studies, we use a newly derived method for FDR correction in combination with data from multiple opioid maladaptation phenotypes to reproduce the earlier *Dcc* association and to identify a novel gene regulating clinically important maladaptations to opioids.

## Results

### Haplotype-based computational genetic mapping of opioid maladaptations

**Fig. 1:**
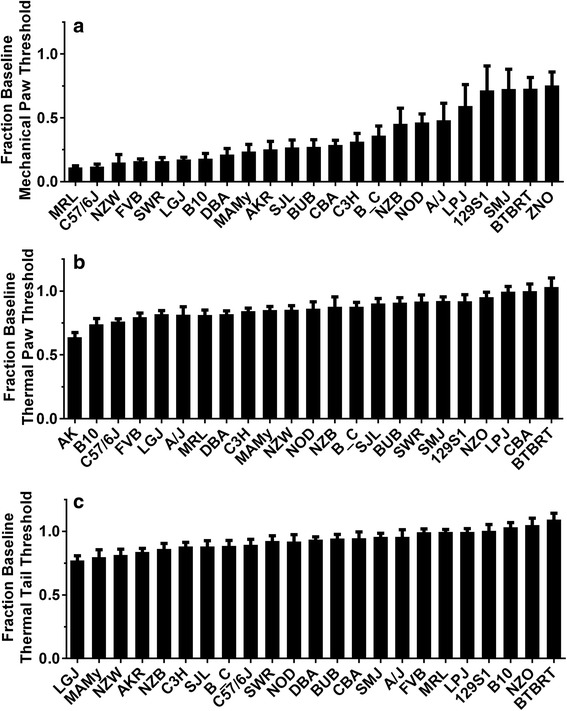
Opioid-induced changes in nociceptive thresholds for 23 strains of mice. These data were reproduced from our earlier report [18]. In panel (**a**) the reduction in mechanical nociceptive thresholds are displayed as a fraction of the baseline thresholds. The strains are displayed from the most to least robustly changed. In panel (**b**) thermal withdrawal thresholds measured using a Hargreaves’ apparatus are displayed as a fraction of baseline threshold. In panel (**c**) changes in thermal withdrawal threshold of the tail (tail flick) response are provided. For each trait the mean value is displayed +/− S.E.M., *n* = 8 mice per strain

**Fig. 2:**
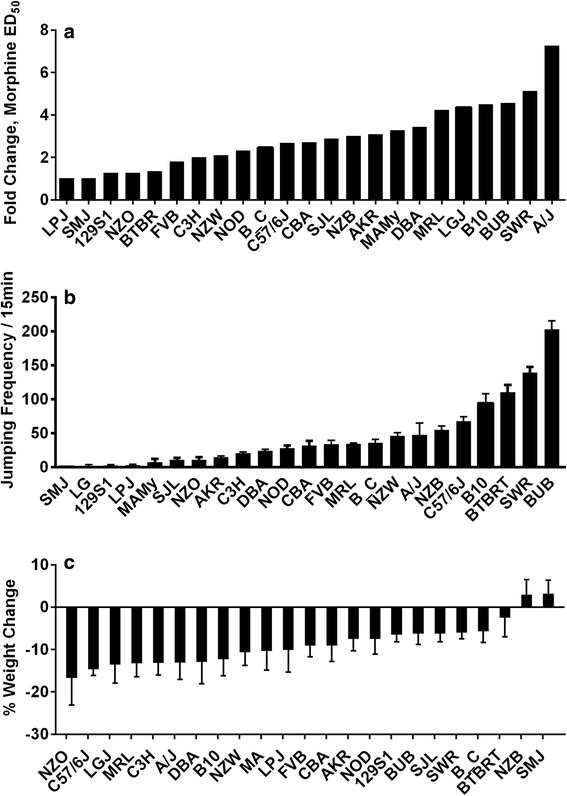
Morphine tolerance, dependence and weight change for 23 strains of mice. Some data were reproduced from our earlier report [18]. In panel (**a**) the fold change in the ED_50_ values for morphine dose–response curves are provided. In panel (**b**) the number of jumps exhibited by the mice in the 15 min period following the administration of naloxone are displayed. In panel (**c**) the change in weight as a fraction of baseline for each of the 23 strains used in these experiments are provided. For each trait the mean value is displayed +/− S.E.M., *n* = 8 mice per strain

Six opiate responses were analyzed in 23 inbred strains, which include: mechanical hindpaw OIH, thermal hindpaw OIH, thermal tail flick OIH, morphine analgesic tolerance, physical dependence and morphine-induced changes in body mass (Figs. 1 and 2). For this analysis, HBCGM was first applied to each phenotype individually. Each analysis produced a list of genes (not corrected for FDR) with patterns of allelic variation that were associated with each phenotype. We then analyzed the results obtained for 6 opioid adaptation phenotypes in an integrated fashion (Table 1). The output of the integrative analysis identifies the genes exhibiting the strongest association to all six phenotypes.

**Table 1:** Rank of genes associated with 6 opioid adaptive phenotypes before FDR correction. While the *Dcc* gene was most strongly associated with the adaptive phenotypes, *Mpdz* was the 26^th^ most strongly associated

Gene	Score
Dcc	26.1
Sgcz	25.4
Sgpp2	25.3
Atrnl1	24.3
Rora	23.6
Lrch1	23.6
Erbb4	23.5
Kank1	23.3
Ctif	23.3
Myo5b	23.3
L3mbtl4	23.2
Setbp1	23.2
Slc9a9	23.0
Dpp10	23.0
Trpm3	22.8
…	…
Mpdz	22.3

In order to correct for false discovery, we applied FDR correction methodology to the individual phenotype mapping results prior to integrating across the phenotypes. The grouped analysis results after FDR and integration are also given in Table 2. Of note, without FDR filtering, *Mpdz* was ranked 26th, whereas after FDR filtering *Mpdz* was ranked 1st. The three phenotypes contributing most strongly to the ranking of *Mpdz* in the FDR corrected mapping results were tolerance, mechanical hindpaw OIH and weight loss. The *Dcc* gene associated with a more limited set of opioid maladaptive traits for these 23 strains and also appears on the final list, though at a lower position than previously found [18]. The *Mpdz* gene has been demonstrated to be involved in synaptic functions including long-term potentiation to which spinally-mediated opioid maladaptations such as tolerance and hyperalgesia have been compared [20, 21]. The *Sgcz* gene listed in second position in Table 1 dropped out of the top 10 most strongly after application of the FDR correction to 60th position, but was still strongly correlated with the physical dependence/jumping phenotype.

**Table 2:** Rank of genes associated the opioid adaptive phenotypes after FDR correction. While the *Dcc* gene remained highly associated, *Mpdz* rose to the top of the list

Gene	Score
Mpdz	14.9
Atrnl1	13.0
Chn2	12.4
En2	12.1
Dcc	11.9
Paqr5	11.7
Slc9a9	10.9
Mup20	10.8
Mkrn1-ps1	10.8
Cyp7b1	10.5
Magi2	10.4
Rps9	10.2
Dck	10.2
Il16	10.2
Rufy3	10.2

The *Mpdz* gene on chromosome 4 codes for the MUPP1 protein. It is greater than 1.6 Mb in length and contains 676 SNPs, which are present in regulatory and coding regions. Ten SNPs alter the predicted amino acid sequence of MUPP1 (Table 3). Of note the alleles at 8 of these SNPs are shared by the 6 strains with highest and lowest levels of opioid adaptation. One allelic substitution (H1767N) occurs at an amino acid position that is conserved across humans and 6 mammalian species. It is located within a PDZ domain in MUPP1 has been shown to be critical for interaction with CaMKII [22], which is a kinase whose spinal activity is critical for the development of tolerance and OIH [23–25].

**Table 3:**
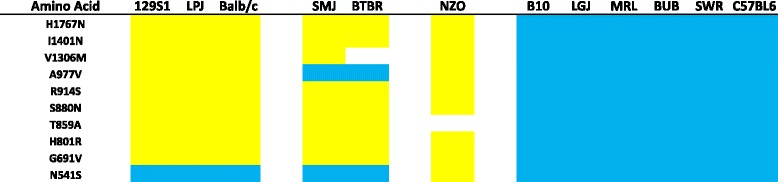
Codon changing SNPs in the murine *Mpdz* gene. Ten variants were identified and are listed in the leftmost column. The table also provides the identity of each of the codon changing SNPs for the six least robustly opioid adapting (left) and six most robustly adapting strains (right). Possession of the common variant is indicated by a blue bar

### *Mpdz* expression in spinal cord tissue

**Fig. 3:**
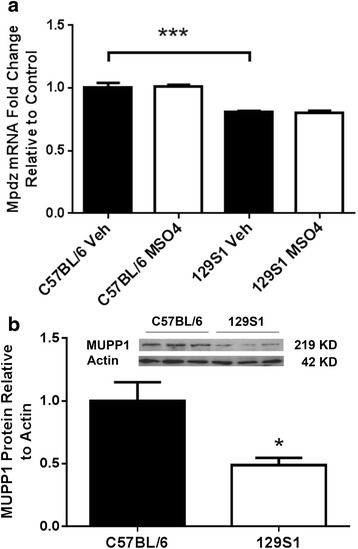
*Mpdz* expression and MUPP1 levels in mouse spinal cord tissue. In these experiments the effects of both genotype and morphine treatment were evaluated. Panel (**a**) provides the results of qPCR experiments in which the index C57BL/6 and low-adapting 129S1/SvIm strains were used. Baseline *Mpdz* levels were lower in the 129S1/SvIm strain, but the levels of *Mpdz* mRNA were unchanged by morphine treatment in both of the strains tested. In panel (**b**) the results of Western analysis are provided demonstrating that 129S1/SvIm mice have lower spinal levels of Mpdz/MUPP1 protein. For each trait the mean value is displayed +/− S.E.M., *n* = 6 mice per group. *p < 0.05, ***p < 0.001

A change in either *Mpdz* expression levels or in the function of the mature MUPP1 protein could contribute to the observed maladaptive differences among the inbred strains. To determine if differences in *Mpdz* expression differed between strains with divergent opioid phenotypes, we examined spinal cord tissue, which a principal center responsible for opioid tolerance and OIH [26]. We selected the C57BL/6 and 129S1/SvIm strains as representative of strongly adaptive and adaptation-resistant strains, respectively. The spinal cord tissue of the C57BL/6 mice had higher levels of *Mpdz* mRNA (Fig. 3a) and protein (Fig. 3b) than the 129S1/SvIm animals. Moreover, morphine treatment did not alter mRNA levels in either strain.

### *Mpdz* knockdown and morphine maladaptations

**Fig. 4:**
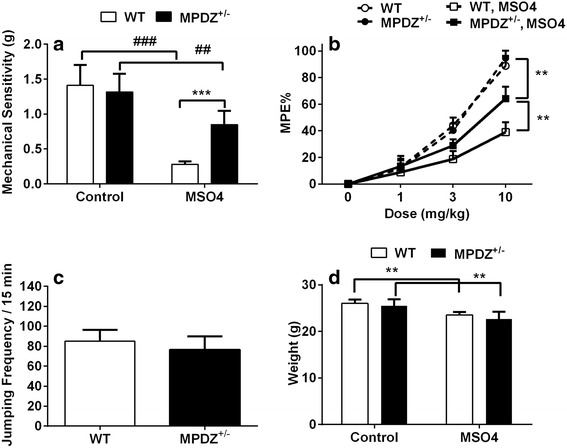
Opioid adaptations in C57BL/6 wild-type and *Mpdz* ^*+/−*^ mice. In panel (**a**) the results of experiments measuring opioid-induced hyperalgesia using changes in mechanical withdrawal thresholds are displayed. In panel (**b**) the anangesic effect of cumulitive doses of morphine are shown for wild-type and *Mpdz* ^*+/−*^ mice both before and after 4 days of escalating dose morphine treatment. In panel (**c**) naloxone-induced jumping behavior for wild-type and *Mpdz* ^*+/−*^ mice is displayed. Finally, in panel (**d**) data for morphine-induced weight loss are provided. For each trait the mean value is displayed +/− S.E.M., *n* = 8 mice per strain. ##, **p < 0.01, ###, ****p < 0.001

In order to test the functional role of *Mpdz* in opioid maladaptations in mice, we tested littermate wild-type and *Mpdz* +/− heterozygous mice in the C57BL/6 background. These heterozygous mice have CNS Mpdz/MUPP1 levels that are 47 % of those found in wild type mice [27]. While the heterozygous mice have normal baseline mechanical nociceptive thresholds, they do not develop OIH to the degree displayed by the wild-type mice (Fig. 4). In addition, analgesic tolerance to morphine is reduced in these heterozygous animals though drug naïve morphine sensitivity was not found to differ between the strains. On the other hand, *Mpdz* heterozygous mice did not display altered physical dependence, nor was the degree of weight loss different after morphine treatment.

### Mpdz/MUPP1 interaction with CaMKII

**Fig. 5:**
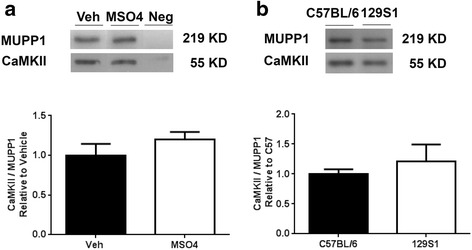
Co-immunoprecipitation of MUPP1 with CaMKII in mouse spinal cord tissue. Spinal cord synaptosomes were first incubated with anti-MUPP1 (or IgG as a negative control). After column purification, the immune complexes were eluted and separated on acrylamide gels. Blots were probed with anti-CaMKII to quantify MUPP1-CaMKII association. Panel (**a**) provides the results of co-immunoprecipitation experiments demonstrating that MUPP1-CaMKII association is unchanged by morphine treatment in C57BL/6 mice. In panel (**b**) the results suggest MUPP1 protein from 129S1/Svlm and C57BL/6 mice bind CaMKII similarly. For each measurement the mean value is displayed +/− S.E.M., *n* = 6 mice per group

Earlier work described the interaction of CaMKII with MUPP1 as a key event governing the activation of NMDA receptors in hippocampal neurons [28]. This has relevance to morphine treatment as both NMDA receptor activation and CaMKII activity in spinal tissue are critical for opioid tolerance and OIH [24, 29]. Co-immunoprecipitation experiments were performed to identify synaptosomal proteins that co-precipitated with MUPP1. After precipitation of MUPP1 protein using an anti-MUPP1 antibody, the amount of associated CaMKII was quantified by immunoblot analysis. Although CaMKII co-precipitated with MUPP1, there was not a difference in the amount of associated CaMKII after morphine treatment in the high adaptive strains (Fig. 5a). Since the 129S1/Svlm strain possesses an amino acid substitution within the CaMKII binding region of MUPP1 (H1767N), we hypothesized we would observe lower CaMKII co-immunoprecipitation with MUPP1 in mouse spinal cord synaptosomes made from 129S1/Svlm animals. Although 129S1/Svlm mice may possess less MUPP1 in their spinal cord tissue, the relative amount of CaMKII co-precipitating with the MUPP1 protein was the same in spinal cord tissue obtained from C57BL/6 and 129S1/Svlm mice (Fig. 5b). Additional experiments quantifying activated (Thr-286) phospho-CaMKII in the MUPP1 immunoprecipitates also failed to identify strain or morphine effects (data not shown).

## Discussion

Despite their undisputed efficacy and wide-spread use for the relief of acute pain, the chronic use of opioids is limited by maladaptations, most importantly tolerance and opioid-induced hyperalgesia (OIH). Dose escalation caused by these maladaptations has been associated with poor functional outcomes, greater rates of overdose and death [6]. Little progress has been made in designing strategies to limit or reverse these problems. Whole-genome genetic mapping studies offer the possibility of identifying new mechanisms underlying key maladaptations that might be targeted using novel treatment strategies. Unfortunately, this form of mapping is prone to high rates of false discovery due to the number of comparisons made in performing whole-genome studies. Our approach of using a false discovery rate (FDR) correction in combination with an integration of mapping results across opioid adaptive phenotypes helps to make the mapping results more reliable. In these studies the *Mpdz* gene was associated with maladaptive opioid phenotypes, and was confirmed as a gene relevant to these phenotypes using *Mpdz* +/− heterozygous mice. This result suggests that we look more closely at the Mpdz/MUPP1 protein and the pathways in which it participates in understanding and treating opioid tolerance and OIH.

A novel aspect of the HBCGM analysis in this study was correcting for the tendency of highly polymorphic genes to show up as being associated with many phenotypes. To address this problem we randomized the mapping from strain names to phenotype measurements many times, and excluded genes that matched too many of the randomized phenotypes. We then ranked genes by a composite score based on their associations with all six related phenotypes. The combination of these methods resulted in the *Mpdz* gene being ranked first, attracting our attention. Using previously described whole genome mapping approaches the *Mpdz* gene was ranked much lower in the list of associated genes (Table 1). On the other hand, a gene previously shown to be highly associated with these phenotypes and confirmed to play a functional role using transgenic animals and biochemical analyses, *Dcc*, remained relatively highly correlated.

It should be realized that additional genetic contributors might be discovered if other measures of OIH were used. We selected a thermal and a mechanical measure to provide some phenotypic diversity and to make the measurement of OIH practical in large numbers of mouse strains. However, models of inflammatory and incisional pain with different mechanisms and transmission pathways also show heightened nociceptive responses after chronic opioid administration [30, 31]. Likewise, a non-thermal stimulus could be used to test analgesic tolerance although most alternatives would be more laborious especially for use in large numbers of animals. Nevertheless, these might be used in separate mapping studies, or at least the genes discovered using simple models could be tested for relevance in other settings.

The MUPP1 protein was originally isolated as a partner of the 5-HT_2C_ receptor [32]. Analysis of the protein revealed 13 PDZ domains thus leading to an alternative name (multi-PDZ-domain protein, Mpdz). A short time later the corresponding murine *Mpdz* gene on chromosome 4 was identified and sequenced [33]; the corresponding protein has a predicted molecular weight of 218 kDa. PDZ domains such as those on MUPP1 represent key structures for protein-protein interaction [34], and are frequently found near sites of cell-cell interaction such as synapses and tight junctions. Indeed several protein partners for MUPP1 have been identified in tight junctions including claudin proteins [35, 36], a somatostatin receptor (SSTR3) [37], and a potassium ion channel (Kir4.2) [38]. Likewise MUPP1 has been localized to synapses in the CNS where the protein is widely expressed [39]. It is the synaptic expression of this gene that may be responsible for the apparent role of Mpdz/MUPP1 in tolerance and OIH. In this regard it is notable that another post-synaptic PDZ domain containing scaffolding protein, PSD95, is also thought to be involved in opioid maladaptations [40, 41].

MUPP1 is a key protein both facilitating the organization of neural synapses and in facilitating synaptic plasticity. Identified binding partners in mammalian synapses for MUPP1 in addition to the originally described 5-HT_2C_ receptor interaction include CADM1 [42], connexin 36 [43], RhoA guanine nucleotide exchange factor (GEF) [44], CaMKII, the GABA_B_ receptor [45], SynGAP [28] and the NMDA receptor subunit NR2B [28]. It has been hypothesized that PDZ-containing proteins such as PSD-95 and MUPP1 may bring intracellular signaling molecules into proximity with cell surface receptors to enhance signaling efficiency. Specifically, MUPP1 brings the NMDA receptor, CaMKII and SynGAP into close post-synaptic proximity [28]. The activation of NMDA receptors then leads to the activation of a MUPP1-facilitated cascade ultimately causing the insertion of AMPA receptor/channels into the membrane, and persistent facilitation of glutamate signaling. This pathway may contribute to phenomena such as long-term potentiation (LTP) or alternative forms of enhanced AMPA receptor mediated activity [46].

Both opioid analgesic tolerance and OIH are reliant upon molecules with which MUPP1 interacts. For example, the pharmacological blockade of NMDA receptors and genetic deletion of NMDA receptor subunits both limit tolerance and OIH after chronic morphine administration in mice and rats [29, 47]. The NR2B subunits of NMDA receptors are specifically involved in morphine tolerance [48, 49]. Likewise, morphine enhances the spinal expression and phosphorylation of CaMKII, and the spinal administration of CaMKII inhibitors reduces the tolerance and OIH displayed in response to chronic morphine administration [24, 25]. Moreover, the enhanced activity of AMPA channels has been demonstrated to play a role in morphine tolerance as well [50, 51]. A central role for MUPP1 in integrating synaptic NMDA-CaMKII-AMPA signaling has been demonstrated in hippocampal tissue [28]. While our results did not demonstrate any obvious effects of MUPP1 sequence variation between two mouse strains with very different propensities to develop opioid maladaptations (129Svlm and C57BL/6), the approximately 50 % lower synaptic levels of MUPP1 in the 129Svlm mice provide a plausible basis for reduced opioid maladaptation by this strain. Consistent with this hypothesis, *Mpdz+/−* mice, that also have about 50 % less CNS *Mpdz* MUPP1 protein [52], demonstrated less tolerance and OIH in our experiments. Thus the mapping result associating *Mpdz* with tolerance and OIH but not basal analgesic potency seems to fit well with our current understanding of maladaptations related to opioid use. Beyond opioids, *Mpdz* has been associated with alcohol and sedative dependence both in mouse and human genetic studies demonstrating that this gene may regulate chronic drug administration effects in multiple classes [53–55].

Much work has been done on the mechanisms supporting tolerance, OIH and other maladaptations to the use chronic use of opioids. These phenomena both limit the clinical utility of the drugs, and place patients at elevated risk of addiction and overdose. Regretably we have no appraoches to delaying the onset or reducing the severity of the maladaptations. Through whole-genome mapping utilizing a new FDR-corrected approach we have demonstrated, however, that novel mechanisms suporting opioid maladaptations can be discovered. We hope that by targeting the protein gene products or perhaps identifying other critical molecules in the involved signaling pathways, we might develop useful therapeutic approaches. In the case of *Mpdz*/MUPP1, the central role of synaptic plasticity is highlighted. Methods of disrupting protein-PDZ interactions, excitatory amino acid signaling or possibly CaMKII activation may be plausible approaches.

## Conclusions

Our results suggest that the *Mpdz* gene helps to control key clinically relevant maladaptations to morphine administration in mice. The basis for the genetic effects may be related to differences in gene expression in spinal cord tissue as levels of *Mpdz* expression varied between mouse strains, but the ability to interact with at least one important binding partner, CaMKII, did not. On the other hand, we have explored only the top-most gene candidate that resulted from our analyses. Other highly ranked genes such as *Atrnl1* or *Chn2* might be pursued as well given there robust association with the maladaptive phenotypes under study. Beyond opioid responses, however, these results suggest whole-genome HBCGM with appropriate FDR correction and the integration of results from the mapping of related phenotypes might provide insight into numerous phenotypes measurable in common strains of inbred mice.

## Methods

### Animal care and use

All experimental protocols were approved by Veterans Affairs Palo Alto Healthcare System Institutional Animal Care and Use Committee prior to beginning the experimentation. Mice were kept under pathogen-free conditions, 4 mice per cage with a 12 h light/dark cycle and an ambient temperature of 22 ± 1 °C. Food and water were available *ad libitum*.

All mice used for the mapping experiments were male and were obtained from The Jackson Laboratory at 11–12 weeks of age, and were kept in our facility a minimum of 1 week prior to use in experiments. The specific strains used to generate the mapping data included 129S1/SvImJ, A/J, AKR/J, B10.D2-H2/n2SnJ, Balb/cJ, BTBR T+ Itpr3tf/J, BUB/BnJ, C3H/HeJ, C57BL/6 J, CBA/J, DBA/2 J, FVB/NJ, LG/J, LP/J, MA/MyJ, MRL/MpJ, NOD/LtJ, NZB/BlnJ, NZO/HlLtJ, NZW/LacJ, SJL/J, SM/J and SWR/J as described previously [18]. *Mpdz* transgenic mice on the C57BL/6 background were obtained from our breeding colony [27]. Littermate wild type and *Mpdz +/−* heterozygous mice were also used at 11–12 weeks of age. All mice used were male, and cohort sizes were 6–8 as noted in the figure legends.

### Chronic morphine administration

After baseline nociceptive testing, morphine sulphate (Sigma) was administered to mice subcutaneously (s.c.) 20 mg/kg twice per day on days 1–3 and 40 mg/kg twice per day on day 4 in 50–100 μl volumes of 0.9 % NaCl. This protocol has been used to demonstrate opioid tolerance, dependence, hyperalgesia and other maladaptive phenotypes in mice in previous experiments [13, 18].

### Behavioral testing – mechanical thresholds

Mechanical withdrawal testing was performed as described previously [14]. Briefly, mechanical nociceptive thresholds were measured using von Frey fibers and the “up-down” algorithm [49]. For these experiments mice were acclimated on wire mesh platforms in plastic cylinders. After acclimation, fibers of sequentially increasing stiffness were applied to the plantar surface of a hind paw according to the up-down paradigm. This testing allowed the estimation of the mechanical withdrawal threshold using curve fitting of the response data [50]. Final measurements were made approximately 18 h after the administration of the final dose of morphine. Mechanical opioid-induced hyperalgesia (OIH) was calculated as the percentage decrease in mechanical nociceptive threshold resulting from repeated morphine administration.

### Morphine dose–response

Cumulative morphine dose–response curves were constructed as described previously [11, 12]. In order to accomplish this, thermal tail flick latencies of gently restrained mice were measured with 0.1 s precision. Two measurements were made per mouse. The lamp intensity was the same for all strains. For the assessment of morphine analgesic tolerance, these dose–response experiments were made on morphine naïve mice and again 18 h after the final dose of morphine was administered. The cumulative doses of morphine used were 0,1,2,4,8,16 and 32 mg/kg s.c. Latency was determined 25 min after morphine administration as demonstrated previously to be the time of peak morphine effect. The parameter %MPE (percent maximal possible effect) was determined according to the following formula:

$$ \%\mathrm{M}\mathrm{P}\mathrm{E}=\left[\left(\mathrm{measured}\ \mathrm{latency}\hbox{-} \mathrm{baseline}\ \mathrm{latency}\right)/\left(\mathrm{cutoff}\ \mathrm{latency}\hbox{-} \mathrm{baseline}\ \mathrm{latency}\right)\right]\mathrm{x}100 $$

### Physical dependence

Physical dependence on morphine was measured using previously reported methods [11, 12]. Mice were treated with morphine for 4 days according to the standard morphine administration protocol described above. To precipitate withdrawal, naloxone (Sigma) 10 mg/kg was injected s.c. in 50 μl 0.9 % NaCl. Mice were then placed in the same clear cylinders used for the mechanical withdrawal threshold assays. The number of jumps made during the 15 min following naloxone injection was counted.

### Weight measurement

Not reported previously for the dataset was the measurement of weight. Animals were weighed individually immediately prior to initiation of testing and morphine treatment using a pan balance accurate to 0.01g. Animals were again weighed after completion of the chronic morphine administration paradigm. To calculate % weight change, the following formula was used:

$$ \left(\left(\mathrm{Final}\ \mathrm{weight}\hbox{-} \mathrm{Initial}\ \mathrm{weight}\right)/\mathrm{Initial}\ \mathrm{weight}\right)\ \mathrm{x}\ 100 $$

### Whole-genome haplotype-based computational genetic mapping

Haplotype-based computational genetic mapping (HBCGM) was conducted as described previously [18, 56], and used to identify those genomic regions whose genetic pattern was correlated with input phenotypic values for the 23 inbred strains utilized in these studies. In our previous work we used tolerance, physical dependence, opioid-induced hyperalgesia to mechanical and thermal hindpaw responses and thermal tail flick data from these 23 strains to discover an association of those traits with the *Dcc* gene [18]. In the present work we used the same 5 datasets and added weight change during morphine exposure. To facilitate computational mapping we converted the nociceptive measurements to the fraction of baseline withdrawal thresholds and latencies, and calculated the fold change in morphine ED_50_ value as done in previous mapping studies [9, 18, 57]. As described below, we also employed a false discovery rate correcting procedure in the present work.

#### Filtering based on gene-wise false discovery rate (FDR) *p* value

Initial studies demonstrated that genes with a large size and/or a high mutation rate (e.g. MHC region genes such as *Skint*) were frequently identified as having a strong association with an analyzed trait. Their large size and/or high rate of polymorphism generated a large number of different haplotypic patterns for these genes, which increased the likelihood that genetic variation within these genes would match a phenotypic response pattern by chance. This problem was addressed using a permutation test to compute a False Discovery Rate (FDR) *p* value for each gene, which is the probability that genetic variation within a gene matches a phenotype by chance. To compute this *p* value, the mapping of strain names to phenotype measurements was permuted 1,000 times, and HBCGM was then run on each of the permuted phenotypes to produce 1,000 *p* values for each gene. The FDR *p* value for each gene was then calculated as the number of *p* values smaller than the original *p* value, divided by the number of permutations (1,000). For any genetic association where the FDR *p* value was greater than 0.005, the gene was removed. This corresponds to an association where the gene has matched randomized data more than five times in the 1,000 permutations.

#### Integration of computational genetic mapping results across related traits

HBCGM was first applied to evaluate each trait. For some analyses, e.g. Table 2, the FDR correction was then applied. To test the strength of association across all opioid traits into account, a score was calculated as described previously [18]:

$$ \mathrm{Score}={\displaystyle \sum_{\mathrm{i}=1}^{\mathrm{n}}}\hbox{-} lo{g}_{10}\left({p}_i\right) $$

In this calculation *i* = 1…n runs through all traits of interest and *p* was defined as described above. In general, genes with multiple highly significant associations have high scores and therefore are of higher interest.

### Protein isolation and western blotting

After euthanization using CO2 asphyxiation, spinal cord tissue was harvested by extrusion. Lumbar spinal cord tissues were dissected on a chilled surface. Tissue was then quick-frozen in liquid nitrogen and stored at −80 °C until the time of analysis. Spinal cords were dissolved at 4 °C in RIPA buffer (50 mM Tris–HCl, pH 8.0, 150 mM sodium chloride, 1.0 % NP-40, 0.5 % sodium deoxycholate, and 0.1 % sodium dodecyl sulfate) to which a protease inhibitor cocktail had been added (Roche). Aliquots of protein were loaded on pre-cast SDS-PAGE (10 % Tris–HCl acrylamide) gels, run and electrotransferred onto a polyvinylidene difluorided membranes [54]. These blots were probed with the primary MPDZ antibody (BD Transduction Laboratories, San Jose, CA) or CaMKII antibody (Abcam) at 4 °C overnight. β-actin (Sigma) was used as an internal control. The band intensity was quantified using National Institutes of Health image J analysis software (version 1.44).

### Spinal cord synaptosome preparation

Spinal cord tissue was homogenized at 4 °C in the Syn-PER Reagent (Thermo Scientific) in the presence of EDTA-free the protease inhibitor cocktail (Roche). The homogenate was centrifuged at 1200 × g for 10 min to remove cell debris, and the supernatant was then centrifuged at 15,000 × g for 20 min. The pellets, containing synaptosomes, were gently resuspended in the Syn-PER Reagent and maintained on ice until use in co-immunoprecipitation experiments.

### MUPP1 co-immunoprecipitation

Co-immunoprecipitation assays were performed using a commercially available co-immunoprecipitation kit according to the manufacturer’s protocol (Thermo Scientific). Briefly, the protein concentration of each isolated synaptosome sample was determined using the Pierce BCA Protein Assay (Thermo Scientific). Equal amounts of synaptosome samples (250 μg) were pre-cleared with control agarose resin at 4 °C for 30 min. The supernatants were then incubated with MUPP1 antibody (BD Transduction Laboratories) or mouse IgG (Cell Signaling) overnight at 4 °C to form immune complexes. To capture the immune complexes, washed protein A/G agarose bead slurry was added into the antibody/lysate samples and incubated for 1 h at room temperature. After centrifugation at 3,000xg for 2 min, the beads were collected and washed with ice-cold lysis buffer three times. Loading buffer was added to the beads which were boiled for 5 min and then centrifuged at 10,000× g for 5 min to dissociate proteins. The co-immunoprecipitated proteins were subsequently analyzed using Western blotting.

### RNA isolation and analysis

Spinal cord samples from separate cohorts of mice were dissected and stored as described above. For real-time quantitative PCR, total RNA was isolated from spinal cord tissue using the RNeasy Mini Kit (Qiagen) according to the manufacturer’s instructions. The purity and concentration were determined spectrophotometrically. Reverse transcription was accomplished using a First Strand complementary DNA Synthesis Kit (Invitrogen). Real-time PCR was performed in an ABI prism 7900HT system (Applied Biosystems). All PCR experiments were performed using the SYBR Green I master kit (Applied Biosystems). All the primers were purchased from SABiosciences (SABiosciences). Quantification was accomplished according to the standard curve method. In order to achieve the same PCR efficiency for each analyte, 1:10 serial dilutions of cDNA were used to construct standard curves for *Mpdz* and GAPDH. The R^2^ values for the standard curves for each analyte approached 1.0, suggesting the same amplification efficiency in the PCR reactions under these conditions. Melting curves were performed to document single product formation and agarose electrophoresis confirmed product size. As negative controls, RNA samples that were not reverse transcribed were run. Data were normalized to GAPDH mRNA expression.

### Statistical analysis for behavioral data

All data are displayed as the means +/− SEM unless otherwise noted. 1- or 2-Way ANOVA analysis was applied to behavioral data with the Tukey test applied post-hoc (Prism 5, GraphPad Software).

### Ethics approvals

All experimental protocols were approved by Veterans Affairs Palo Alto Healthcare System Institutional Animal Care and Use Committee prior to beginning the experimentation.

### Availability of data and material

The datasets supporting the conclusions of this article is are included within the article.

## Abbreviations

AMPA: α-amino-3-hydroxy-5-methyl-4-isoxazolepropionic acid
CaMKII: calmodulin-dependent protein kinase II
CDC: centers for disease control
Dcc: deleted in colorectal cancer
FDR: false discovery rate
GABA: gamma-aminobutyric acid
GEF: guanine nucleotide exchange factor
HBCGM: haplotype-based computational genetic mapping
Mb: megabase
MPDZ: multiple PDZ-containing protein
MUPP: multiple PDZ-containing protein (alternate acronym)
NMDA: N-methyl-D-aspartate
OIH: opioid-induced hyperalgesia
PDZ: post synaptic density protein, drosophila disc large tumor suppressor, zonula occludens-1 protein
PSD95: post synaptic density protein 95
QTL: quantitative trait loci
Sgcz: sarcoglycan zeta
SynGAP: synaptic ras GTPase activating protein
US: United States
